# The Electrical Conductivity and Mechanical Properties of Monolayer and Multilayer Nanofibre Membranes from Different Fillers: Calculated Based on Parallel Circuit

**DOI:** 10.3390/polym14225048

**Published:** 2022-11-21

**Authors:** Lijian Wang, Chuanhe Li, Jishu Zhang, Chunhong Wang, Qi Zuo, Wenting He, Ligang Lin

**Affiliations:** 1 School of Textile Science and Engineering, Tiangong University, Tianjin 300387, China; 2 School of Textile, Garment & Design, Changshu Institute of Technology, Changshu 215500, China; 3 School of Materials Science and Engineering, Tiangong University, Tianjin 300387, China

**Keywords:** electroconductive fillers, interfacial effects, parallel resistance

## Abstract

Advanced research on improving the performance of conductive polymer composites is essential to exploring their potential in various applications. Thus, in this study, the electrical conductivity of multilayer nanofibre membranes composed of polyvinyl alcohol (PVA) with different electroconductive fillers content including zinc oxide (ZnO), multiwalled carbon nanotubes (MWNTs), and Ferro ferric oxide (Fe_3_O_4_), were produced via electrospinning. The tensile property and electrical conductivity of monolayer membranes were explored. The results showed that PVA with 2 wt.% MWNTs nanofibre membrane has the best conductivity (1.0 × 10^−5^ S/cm) and tensile strength (29.36 MPa) compared with other fillers. Meanwhile, the combination of multilayer membrane ZnO/Fe_3_O_4_/Fe_3_O_4_/MWNTs/ZnO showed the highest conductivity (1.39 × 10^−5^ S/cm). The parallel circuit and calculation of parallel resistance were attempted to demonstrate the conductive mechanism of multilayer membranes, which can predict the conductivity of other multilayer films. The production of multilayer composites that enhance electrical conductivity and improve conductive predictions was successfully explored.

## 1. Introduction

Electrospinning is an efficient method of producing nanosized or micro-sized fibres using an electrical field. When millions of fibres are collected on the collector device or rotating disk collector, dry polymer fibres of submicron diameter were deposited and formed nanofiber membranes [1,2]. In the electrospinning process, nanofibres are formed by driving the polymeric jet from the premixed solution under high voltage [3,4]. The pre-mixed solution usually consists of a polymer with a volatile solvent and different nanofillers. The incorporation of nanofillers in the electrospun nanofibrous membrane has been used to increase the nanofibre membrane functionality, such as to improve the mechanical, electrical, and thermal properties [5]. The functional membrane has broadened its applications, such as infiltration, adsorption, wound dressing, drug delivery, protective textiles, sensors, and batteries [6,7,8]. 

Textile material that contributes to the electrostatic effect due to friction is not suitable to be worn or used near explosive surroundings, such as at gas stations or explosive handling areas because there could be a high risk of explosion [9]. Therefore, exploring protective textiles without electrostatic effect is necessary to protect personnel when coming into contact in high-risk areas. For conductive function in antistatic applications, the electrical performance of textile can be modified by surface coating, electrospinning, and other methods. In electrospinning, the use of antistatic chemicals, metallic particles, metallic compounds, carbon-based fillers, or inherent conductive polymers can offer antistatic properties to the nanofibrous membrane [10,11]. 

PVA is water-soluble and is easy to form nanofibers by electrospinning, and it has extensive applications due to its biocompatible and biodegradable properties [12]. It is well understood that pure PVA nanofibres are nonconductive materials. A standard method of preparing a conductive nanocomposite is to add electroconductive fillers such as graphite, carbon nanotubes, ZnO, or Fe_3_O_4_ into the polymer matrix [13,14,15,16]. Incorporating conductive fillers into PVA nanofibres enhances the electrical properties of materials compared with nonconductive material consisting of pure PVA nanofilm. Srikanth and co-researchers used PVA mixed with ZnO to prepare the nanocomposite films by solution casting, and maximum conductivity was found at 1 wt.% of ZnO [14]. This demonstrated that ZnO endowed PVA-ZnO nanocomposite films with perfect electroconductivity based on the conductivity of the embedded metallic compounds. Ramos and co-researchers showed that the conductivity of MWCNT/PVA nanofibres by electrospinning increased as the MWCNT content increased [16,17]. With the MWCNTs added into the PVA with the assistant of the electric field, conductive meshes of the nanofibre membrane formed. 

However, most of the electrospun nanofibre membrane studies focus on the production of monolayer and single-added particles or monolayer and multicomponent fillers. Multilayer nanofibre membranes are a common form of arrangement in composites that contribute to outstanding material performance compared with monolayer composites [18]. Multilayer and multivariate nanofibre membranes could be a new approach to improving the conductive performance of electrospun nanofibre membranes. Chen and co-researchers tested a four-point probe for characterizing the resistivity of monolayer or multilayer films that were produced by depositing similar materials on each layer [19]. A multilayer film can be modelled as a simple parallel equivalent circuit with multiple resistances affected by a wide range of resistivity and thickness ratios. Nevertheless, by increasing the number of layers in the composite material and replacing the fillers in the membrane, the conductive pathways of parallel interfaces tend to break apart or change [20]. 

In this paper, we present the electrical conductivity of multivariate and multilayer nanofibre membranes using an improved formula to calculate in parallel circuits by considering the interfacial effects between the adjacent films. Before that, the optimization of different filler contents, tensile properties, and electrical conductivity of the monolayer nanofibre membranes were investigated. A hot-pressing process then prepared the multivariate and multilayer nanofibre membranes with three optimal nanofibre membrane contents (wt.%) following a particular combination.

## 2. Materials and Methods

### 2.1. Material

Polyvinyl alcohol (PVA) (No. 217, molecular weight: 72,600∼81,400, viscosity 20.5∼24.5 × 10^−3^ centipoises) was obtained from Guangzhou Feng Bai Shun Trade Co., Ltd., Guangzhou, China; nanosized zinc oxide (ZnO) (100 nm) was supplied by Shijiazhuang Baisheng Chemical Co., Ltd., Shijiazhuang, China; nanoscale magnetite or ferro ferric oxide (Fe_3_O_4_) (80 nm) were provided by Qinhuangdao Taiji Ring Nanoscale Co., Ltd., Qinhuangdao, China); multiwalled carbon nanotubes (MWNTs) (9.4∼16.4 nm) purchased from Qinhuangdao Taiji Ring Nanoscale Co., Ltd., China. 

### 2.2. Production of Monolayer Nanofibre Membrane via Electrospinning

The PVA polymer was dissolved in distilled water (PVA: distilled water = 1:9), and the mixture was stirred for 12 h at room temperature. The PVA solution was defoamed and loaded into a 15 mL syringe (needle number 21) for the electrospinning. Different electroconductive fillers (ZnO, Fe_3_O_4_, and MWNTs) and mass fractions (0 to 5 wt.%) were prepared, as shown in Table 1. The PVA and filler solution were simultaneously sprayed at the tip collector with a distance of 25 cm, a voltage of 18 kV, and a feeding rate of 0.5 mL/h. The rotating (200–300 rpm) drum was covered with aluminized paper to collect the formed nanofibres. The formed nanofibre membrane was left to dry at 50 °C for 12 h.

### 2.3. Preparation of Multilayer Nanofibre Membrane

The dried PVA/ZnO, PVA/Fe_3_O_4_, and PVA/MWNTs nanofibre membranes were carefully removed from the aluminized paper and laminated. The combinations of multilayer nano-film membranes were prepared, as shown in Table 2, via hot pressing.

### 2.4. Characterization of Monolayer and Multilayer Nanofibre Membranes

Before testing, the laminated membranes were placed in a constant temperature and humidity laboratory for 24 h to reach equilibrium. The thickness was first measured, and then the samples were prepared with circle diameters of 20 mm each. The electroconductivity of membranes was analysed using broadband dielectric spectroscopy (BDS50, Novocontrol Technologies Co., Ltd., Montabaur, Germany). The mechanical properties (tensile strength and modulus) of the membrane were then measured with a universal strength testing machine (3369, US INSTRON Company). Five tests for each group of samples (90 mm (length) × 10 mm (width)) were performed under stoke control at a constant speed of 10 mm/min, and the width of clamps was 30 mm. The optical images for all fabricated nanofibre membranes of PVA with different fillers were observed under a scanning electron microscope (SEM) (TM-3030, Hitachi, Ltd., Tokyo, Japan).

## 3. Results and Discussion

### 3.1. Production of PVA Monolayer Nanofibre Membranes with Different Electroconductive Fillers (ZnO, Fe_3_O_4_, and MWNTs) 

**Figure 1:**
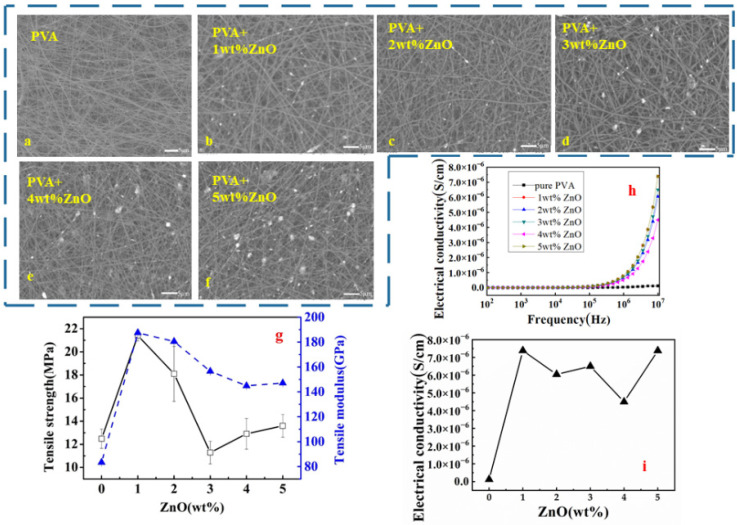
The performance of PVA/ZnO membrane in different mass fractions: (**a**–**f**) the surface of the membrane by SEM; (**g**) the tensile strength and modulus of membrane; (**h**,**i**) the conductive property of membrane in different frequencies.

Figure 1 shows the micrograph PVA/ZnO hybrid-fibre membrane at different mass fractions (1 to 5 wt.%). Based on the SEM micrographs shown in Figure 1a–f, as the ZnO content increased, the distribution of filler particles within PVA fibre showed a nonhomogenous distribution followed by reunion and agglomeration at a high percentage of filler content. On the electrospun nanofibre membrane at 1 wt.%, the ZnO particles were uniformly distributed, while at 2 wt.%, the ZnO particles were randomly scattered throughout the PVA nanofibre membrane as displayed in Figure 1b,c, respectively. This suggested that at 1 and 2 wt.%, ZnO was successfully embedded in the one-dimensional hybrid fibres via chemical interactions between ZnO and PVA [21]. Sui and co-researchers demonstrated the existence of interactions of PVA/ZnO hybrid fibre via the hydrogen bond formation between the PVA molecule and O–Zn–O in ZnO [21]. However, at 3 and 4 wt.%, the agglomeration of ZnO particles started to occur, which could be observed within the PVA fibre; at 5 wt.%, the agglomeration of ZnO with bulk formation was noticeable (Figure 1d–f). This suggested that more ZnO particles could not readily disperse or embed within the PVA fibre; consequently, agglomeration outside the PVA matrix fibre occurred. Particle agglomeration can be avoided by inducing a high electric field to increase the polarization of the ZnO particles, which then influences the attractive and repulsive interactions between the particles [22].

**Figure 2:**
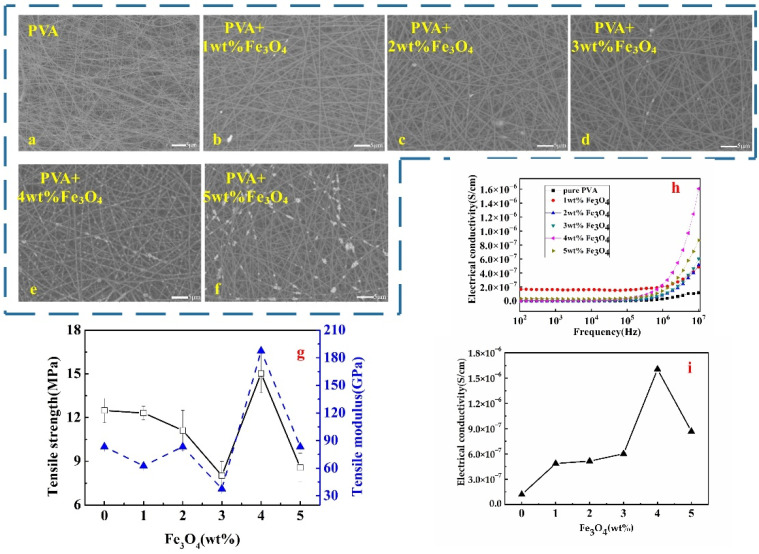
The performance of PVA/Fe_3_O_4_ membrane in different mass fractions: (**a**–**f**) the surface of the membrane by SEM; (**g**) the tensile strength and modulus of membrane; (**h**,**i**) the conductive property of membrane in different frequencies.

On the other hand, a different pattern of particle distributions was shown by the PVA nanofibre membrane with Fe_3_O_4_ (Figure 2). A homogenous particle distribution of magnetite particles was observed for electrospun PVA with 1 to 3 wt.% of Fe_3_O_4_ fillers; the particles were almost invisible on the matrix fibre, as seen in Figure 2b–d. This explained the success of Fe_3_O_4_ particles embedded into the nanofibre membrane, which could have been due to the smaller size of the particle (80 nm) of Fe_3_O_4_ compared with ZnO (100 nm). Wang and co-workers explored the in situ composite approach of electrospun PVA/Fe_3_O_4_ and found that PVA acted as a stabilizer during the coprecipitation [15]. Thus, during the electrospinning process, PVA stabilized Fe_3_O_4_ by making it compatible and embedded together within the fibre. Hence, less precipitation formed on the matrix fibre. However, an agglomeration of fillers appeared on the nanofibre surface at 4 wt.%, uniformly attached to the matrix fibre as shown in Figure 2e. Comparable results were obtained that at 4 wt.% content, Fe_3_O_4_ particles were agglomerated and had attached to the nanofibre surface [15]. Nonuniform particle agglomeration was predominantly observed on the matrix fibre for the PVA hybrid at 5 wt.% Fe_3_O_4_ (Figure 2f). The higher Fe_3_O_4_ mass fraction used in the electrospun PVA induced particle agglomeration, which could have influenced its mechanical, physical, and conductivity properties.

**Figure 3:**
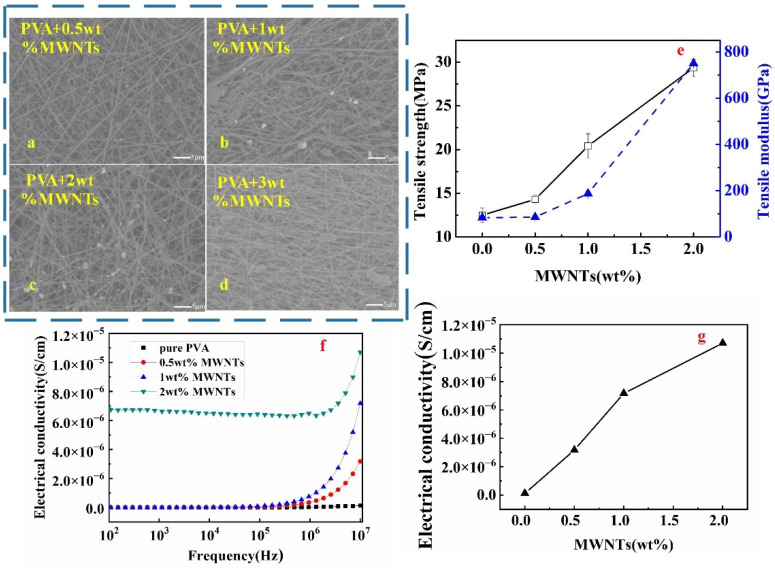
The performance of PVA/MWNT membrane in different mass fractions: (**a**–**d**) the surface of the membrane by SEM; (**e**) the tensile strength and modulus of membrane; (**f**,**g**) the conductive property of membrane in different frequencies.

Lower mass fractions of PVA/MWNTs (0.5, 1, 2, and 3 wt.%) were used, and the nanofibre membrane micrographs are shown in Figure 3a–d. The nanotube-polymer composite preparation was predominantly affected by filler dispersion, orientation, and interfacial bonds within polymers [23]. Similar to the PVA nanofibre membrane with Fe_3_O_4_ and ZnO, the results showed that the MWNT particles were not observed on the PVA matrix fibre when the MWNT content was 0.5 wt.% (Figure 3a). It can be assumed that all MWNT particles successfully embedded in the PVA fibre due to the smaller particle size of MWNT, around 9.4∼16.4 nm; this made it easy to spin and distribute evenly within the PVA. Meanwhile, at 1 to 2 wt.%, MWNT particles were uniformly dispersed and embedded in the PVA fibre, with some of the MWNTs observable on the PVA nanofibre as seen in Figure 3b,c. Other studies showed similar findings that adding lower concentrations of MWNTs produced smooth, undistorted fibre surfaces that were attributed to the fine dispersion of MWNTs within PVA composite fluid [24]. The flaky MWNT aggregation was found on the PVA matrix surface, as shown in Figure 3d. This is probably due to the large van der Waals force, huge specific surface area, and very high aspect ratio between carbon nanotubes. It is proposed that MWNTs entangled and blocked the electrospinning syringe when the MWNTs content was 3 wt.%, resulting in a failure to collect on the PVA/MWNT membrane. Research showed that the polymer-spun fibres containing 5 to 7.5 wt.% MWNTs caused defects in the nanofibre with the formation of notches or beads attributed to agglomeration of MWNTs in PVA solution [24]. Thus, a rougher nanofibre membrane was formed at a higher mass fraction consisting of local nonuniformities, notches, and beads [24].

### 3.2. Tensile Properties of Monolayer Nanofibre Membrane with Different Electroconductive Fillers (ZnO, Fe_3_O_4_, and MWNTs)

The tensile properties of PVA/ZnO nanofibre membrane with varying mass fractions are shown in Figure 1g. A significant increment of tensile strength from 12.49 to 21.41 MPa was observed when the ZnO content increased from 0 to 1 wt.%. The 2 wt.% PVA/ZnO membrane showed 44.92% higher than pure PVA membrane, but 15.4% lower than 1 wt.%. Results were in a parallel pattern with particle distribution within PVA fibre, where at 1 wt.%, ZnO particles uniformly distributed and randomly scattered at 2 wt.%. It is indicated that the filler dispersed well and showed excellent compatibility between PVA and ZnO, which enhanced the tensile strength and Young’s modulus of the membrane [25]. Rueda and co-workers reported that the rigidity of the film increases was due to strong interaction between filler (ZnO) and matrix (PVA) and consequently restricted the motion of the matrix [26]. However, when ZnO particles increased from 3 to 5 wt.%, significant reductions of tensile strength and modulus were observed. Similarly, decrements of tensile strength and modulus were reported due to low interparticle interactions (PVA/ZnO), which create the weakest point in the PVA matrix [25]. The tensile modulus of membranes at 2 to 5 wt.% ZnO fillers was still higher than pure PVA membrane, which indicated that the ZnO fillers could improve the tensile modulus even after agglomeration. Metal oxide particles can reinforce the electrospun film [27], but due to the particle agglomeration phenomenon, these negatively influenced the load transfer of the membrane. That was why the tensile properties increased first and then decreased. As for the modulus results, the tensile modulus and elastic modulus are related to the physical properties of the material itself. This means that the addition of metal oxide (ZnO) successfully changed the mechanical property of the monolayer PVA nanofibre membrane. 

The fluctuation results for the tensile properties of the PVA/Fe_3_O_4_ nanofibre membrane are exhibited in Figure 2g. It is evident that the tensile strength and modulus gradually decreased from 0 to 3 wt.%, followed by a significant increment with the highest at 4 wt.% Fe_3_O_4_ and a sudden drop at 5 wt.%. This tensile pattern occurred probably due to the cohesion force that weakened and slippage of Fe_3_O_4_ particle among fibres PVA matrix; thus, decrement of tensile strength was observed at the first 1 to 3 wt.%. Meanwhile, at 4 wt.% Fe_3_O_4_, the particle cohesion force increased suddenly, similar to the tensile strength and tensile modulus with 15.03 MPa and 187.50 GPa, which increased by 20.26 and 125.01%, respectively, in comparison with the pure PVA membrane [15]. However, too many Fe_3_O_4_ fillers (more than 5 wt.%) resulted in particle agglomeration, which led to the decrease of its tensile properties. This result is in accordance with previous research, which reported at 1 to 3 wt.% Fe_3_O_4_ loading exhibit no significant difference on tensile stress and ultimate strain; however higher value was observed if more than 5 wt.% [28]. The non-uniform distribution, no specific arrangement of conductive particles within the PVA matrix failed to increase its tensile strength. Again, this exemplified that uniform distribution filler within the matrix is an important factor influencing the membrane tensile properties. 

Interestingly, a different pattern of tensile strength and modulus of PVA/MWNTs membrane was observed with continuous increment as the mass fraction of MWNTs fillers (0 to 2 wt.%) increased. Jeong and co-researchers also reported the increment of tensile strength at 1 to 2.5 wt.% MWNTs, which attributed to a higher degree of filler orientation in the wrap nanofibre [24]. The previous study reported the enhancement of tensile properties at lower wt.% of MWNTs filler due to the microchemical interlocking, non-uniform chemical bonding, and weaker van der Waals bonds between MWNTs and PVA matrix [24]. The maximum tensile strength and modulus of the membrane were achieved with the 2 wt.% MWNTs is 29.36 MPa and 750 GPa, which significantly improved by 135.26% and 800%, respectively, compared to that of pure PVA. However, it was difficult to spin when MWNTs exceeded 2 wt.%. It is probably due to the agglomeration of MWNTs, which simultaneously decreased its anisotropy degree [24]. Due to the excellent stiffness and strength of MWNTs, MWNTs are the ideal nanofiller candidate for the reinforcement of nanocomposites [29]. Compared to all three fillers, PVA/MWNTs possess greater tensile strength, and modulus corresponded to better mechanical properties of nanofibre membrane.

### 3.3. Electroconductivity Performance of Monolayer Nanofibre Membrane with Different Electroconductive Fillers (ZnO, Fe_3_O_4_, and MWNTs)

The conductivities of monolayer nanofibre membranes of PVA/ZnO with different wt.% of ZnO (1 to 5 wt.%) at different frequencies (10^2^ to 10^7^ Hz) are shown in Figure 1h. There was no significant increment in conductivity observed from 10^2^ to 10^5^ Hz. Meanwhile, at approximately 10^6^ Hz, the increment was more obvious where the conductivity exponentially increased until 10^7^ Hz for all samples. Thus, from this finding, the conductivity of monolayer nanofibre membrane of PVA/ZnO was fixing at 10^7^ Hz frequency to investigate the effects of different wt.% of ZnO (1 to 5 wt.%) as interpreted in Figure 1i. The coincident conductivity curves of 1 to 5 wt.% ZnO contents can be observed in Figure 1h,i. At 10^7^ Hz, the coincident conductivity increased when the filler content was at 1 wt.% and decreased for higher filler contents. However, when the ZnO filler was at 5 wt.%, the conductivity was as higher as 1wt.%. In combination with the SEM micrograph, the uniform distribution of 1 wt.% ZnO in PVA fibrous membranes contributing to the possibility of forming a conductive pathway among the particles. The higher amount of ZnO fillers caused severe agglomeration, owing to the higher content and large specific surface area and interaction force of nanosized particles. In contrast, filler at 5 wt.% was enough to contribute to the conductive pathway. The optimization of ZnO contents in the PVA matrix was at 1wt.% because of its sharp efficiency effect; thus, it was referred to as threshold. In particular, the relative conductivity effect should enormously increase when the concentration of filler particles approaches the conductive threshold [30]. Therefore, it can be concluded that at 1 wt.% ZnO filler revealed as the best performance both on the tensile and the conductivity properties.

The conductivities of monolayer nanofibre membranes with different Fe_3_O_4_ mass fractions at different frequencies (10^2^ to 10^7^ Hz) are illustrated in Figure 2h, and the conductivities at 10^7^ Hz are displayed in Figure 2i. Figure 2h showed that the conductivity at 4 wt.% Fe_3_O_4_ fillers was the highest from 10^6^ to 10^7^ Hz. The curve of all samples in Figure 2i has a similar trend as in Figure 1h. Therefore, it is reasonable to speculate that the conductivity may be associated with the filler distributions in monolayer nanofibre membrane. Homogenous distribution of Fe_3_O_4_ filler played an important role in forming the conductive network. Hence, with prominent particle cohesion force at 4 wt.% Fe_3_O_4_, it can be determined that the conductivity threshold and the optimized content for PVA/Fe_3_O_4_ were at 4 wt.%.

Figure 3f shows that electrical conductivities of PVA/MWNTs monolayer nanofibre membranes with different MWNTs mass fractions at different frequencies (10^2^ to 10^7^ HZ). Similar to PVA/ZnO and PVA/Fe_3_O_4_ monolayer membranes as showed in Figure 1h and Figure 2h respectively, the conductivity of PVA/MWNTs as displayed in Figure 3f exponentially increased at approximately 10^6^ Hz until 10^7^ Hz. The continuous increasing trend with the increase wt.% of MWNTs is portrayed in Figure 3g. The higher content of MWNTs, allows further individual conductive nano-granules; thus, the conducting bridged structure is easier to form, which has been advantageous to the conductivity of the membrane [16]. The continuous network made by individual carbon nanotubes will act as a conductive path for electrons and make the membrane electrically conductive [31]. The conductive property at 2 wt.% MWNTs membrane has a comprehensive improvement even in low frequency (6.0 × 10^−6^). The PVA membrane obtained conductive property at 2 wt.% MWNTs due to the conductive nature of carbon nanotubes also contributed to a decrease in internal resistance [32]. Finally, considering mechanical and conductive properties, the optimum spinning mass fractions of electroconductive fillers of ZnO, Fe_3_O_4_, and MWNT in films were 1, 4, and 2 wt.%, respectively.

### 3.4. Preparation of Multilayer Nanofibre Membranes and Analysis of Electroconductivity Performance

**Figure 4:**
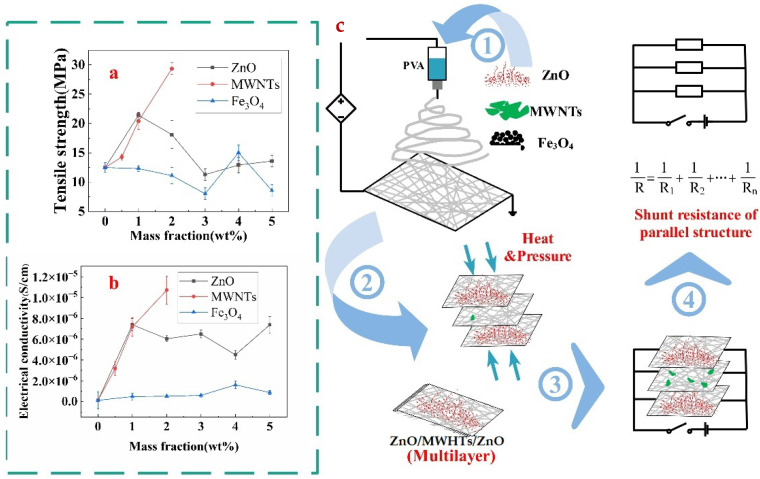
The comparison of different properties of monolayer membranes: (**a**) Tensile strength; (**b**) electrical conductivity at 10^7^ HZ frequency and (**c**) the production of multilayer nanofibre membranes used by optimal single-layer membranes.

The tensile strength and electrical conductivity comparisons at 10^7^ Hz frequency of membranes among different fillers are presented in Figure 4a,b, respectively. The maximum tensile strength for the PVA nanofibre membrane was at 2 wt.% MWNTs. The result revealed that the reinforcement effect of MWNTs on the film was palpable. The tensile strength at 1 wt.% ZnO was at a moderate level. The mechanical property increased suddenly due to the uniform distribution of 4 wt.% Fe_3_O_4_ particles in the PVA matrix. Despite this, the lowest result in tensile strength was observed when Fe_3_O_4_ filler was used in the membrane. The electrical conductivity at 10^7^ Hz frequency showed a similar trend as tensile strength, which is that the sequence from higher to lower conductivity at 2 wt.% MWNTs, 1 wt.% ZnO and 4 wt.% Fe_3_O_4_ membrane. Therefore, the PVA membrane with ZnO was selected to be the surface layer. The MWNTs membrane with the highest conductivity and the lowest Fe_3_O_4_ membrane was utilized as the interlayers for more effective comparison.

**Figure 5:**
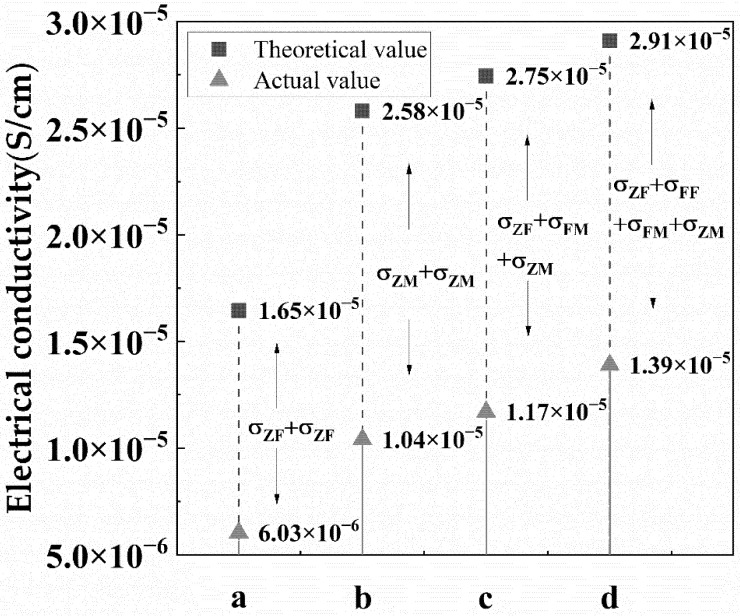
Electrical conductivities of multilayer membranes in the theoretical value and actual value.

Multivariate and multilayer nanofibre membranes were produced by different univariate and monolayer electrospun nanofibre membranes through the heat and press process, as presented in Figure 4c. Figure 5 indicated that the electrical conductivity of ZnO/MWNTs/ZnO was better than that of ZnO/Fe_3_O_4_/ZnO due to the contribution of higher electrical conductivity of PVA/MWNTs. The four-layer membranes were prepared with two interlayers (Fe_3_O_4_ and MWNTs). Finally, the additional Fe_3_O_4_ was added to the four-layer membranes. Figure 5 shows that the increase in conductivity was consistent through the theoretical calculation and actual experimental values. Better conductivity was observed when more layers were used. Although the inter-layer Fe_3_O_4_ membrane possessed lower conductivity, however, the effect on the conductivity of the multilayer membrane was greater than that of the single membrane itself.

The parallel circuit was drawn into a multilayer membrane, shows in Figure 4c. Every membrane was regarded as electric resistance; thus, the rule of shunt resistance could explain the electrical conductivity of multilayer membranes. In theory, the resistance of the multilayer is calculated by Equation (1).

1/R = 1/R_1_ + 1/R_2_ +…+ 1/R_n_ (1)

where R is the total resistance of multilayer material, R_n_ is the resistance of different monolayer membrane. According to R = *ρ*L/S and *ρ* = 1/σ, the total electrical conductivity of multilayer membranes could be calculated by Equation (2).

σ = σ_1_ + σ_2_+…+ σ_n_ (2)

where σ is the total electrical conductivity of the multilayer membrane, σ_n_ is the electrical conductivity of different monolayer membrane. 

However, incompatibility or interaction between adjacent layers was inferred from the interfacial effects between different conductive films and the diverse conductive mechanisms of different conductive particles. The result from the calculation showed the conductivity of the actual multilayer films was considerably lower than the value calculated by Equation (2). The revised equation (Equation (3)) for the total electrical conductivity of the multilayer membrane was put forward, which revealed the regularity of interfaces.

σ = σ_1_ + σ_2_ +…+σ_n_ − (σ_1 2_+σ_2⋅3_+…+ σ_n−1⋅n_) (3)

where σ_n−1⋅n_ is the lost conductivity due to the incompatibility of between adjacent membrane. If the two adjacent films have similar conductive particles, the σ_n−1⋅n_ is assumed to be zero. The lost conductivity of each adjacent film could be calculated. Finally, the σ_ZF_,σ_ZM_, and σ_FM_ were worked out through the theoretical value and actual value. The predicted actual electrical conductivity of the ZnO/Fe_3_O_4_/Fe_3_O_4_/MWNTs/ZnO membrane based on Equation (3) was 1.335 × 10^−5^ S/cm, and the error value was 3.96%, lower than 0.05. Therefore, Equation (3) was considered able to predict the multivariate and multilayer membranes. 

Finally, according to the revised equation, the conductivity of multivariate and multilayer conductive membranes relies on the number of layers, the conductivity of different single films, and the interfacial effects between adjacent films. The characteristic of parallel circuits is that the total resistance is smaller than the minimum parallel resistance. The total resistance decreases when the new resistance is added [33,34], thus with the increase in the number of layers, the conductivity of the system also increases. Different conductive fillers are used to produce membranes, and then the multivariate membranes are composed of different kinds of single films. The electrical conductivity of the membrane with MWNTs is better than Fe_3_O_4_, so the electrical conductivity of ZnO/MWNTs/ZnO is better than ZnO/Fe_3_O_4_/ZnO because the decreasing degree of resistance is related to the added resistance in parallel circuits [34]. When a more effective conductive membrane is added into the system, the conductivity of the multilayer membrane is higher. Interfacial effects are deduced to exist between adjacent but different films. The rule of parallel circuits is applied to adjacent films with the same filler, regarded as the same film of varying thickness. Chen and co-researchers found that the multilayer film with the same filler is modelled as an equivalent circuit with multiple resistances connected in the parallel circuits in a certain thickness range [13]. However, Figure 5 shows that adjacent films with different fillers have an incompatible problem, which results in interfacial effects on attenuating the electron transfer. Therefore, after considering the interfacial effects, the total resistance is higher than the theoretical resistance in the multivariate and multilayer system, meaning that the theoretical conductivity is greater than the actual one. However, different combinations of films can give different properties to multilayer films. For example, the film with Fe_3_O_4_ has electromagnetic shielding effectiveness [35], and the surface layer with ZnO filler has an anti-ultraviolet effect and bactericidal effect [36,37]. The application range of multivariate and multilayer conductive membranes in the protective textile field will be huge in the future. This section may be divided by subheadings. It should provide a concise and precise description of the experimental results, their interpretation, as well as the experimental conclusions that can be drawn.

## 4. Conclusions

Conductive PVA-based nanofibre membranes with different electroconductive fillers (ZnO, Fe_3_O_4_ and MWNTs) were successfully produced by electrospinning. A PVA/MWNTs monolayer membrane particularly for 2 wt.% was well dispersed and uniformly embedded within PVA fibre, resulting in the highest tensile strength (30 MPA) and revealing great electrical conductivity (1.0 × 10^−5^ S/cm). The multilayer film conductivity was successfully estimated using the modified conductivity formula based on parallel circuits. However, the accuracy of the lost conductivity of each adjacent film mentioned in this paper still needs to be investigated.

## Figures and Tables

**Table 1:** Mass fractions of different electroconductive fillers.

Type of Electroconductive Fillers	Mass Fractions of Electroconductive Fillers (wt.%)
ZnO	1, 2, 3, 4, 5
Fe_3_O_4_	1, 2, 3, 4, 5
MWNTs	0.5, 1, 2, 3

**Table 2:** The combination of the multilayer membrane.

Number	Rules of the Multilayer Membrane
a	ZnO/Fe_3_O_4_/ZnO
b	ZnO/MWNTs/ZnO
c	ZnO/Fe_3_O_4_/MWNTs/ZnO
d	ZnO/Fe_3_O_4_/Fe_3_O_4_/MWNTs/ZnO

## Data Availability

Not applicable.
